# PTBP1-associated microRNA-1 and -133b suppress the Warburg effect in colorectal tumors

**DOI:** 10.18632/oncotarget.8005

**Published:** 2016-03-09

**Authors:** Kohei Taniguchi, Miku Sakai, Nobuhiko Sugito, Minami Kumazaki, Haruka Shinohara, Nami Yamada, Tatsushi Nakayama, Hiroshi Ueda, Yoshihito Nakagawa, Yuko Ito, Manabu Futamura, Bunji Uno, Yoshinori Otsuki, Kazuhiro Yoshida, Kazuhisa Uchiyama, Yukihiro Akao

**Affiliations:** ^1^ United Graduate School of Drug Discovery and Medical Information Sciences, Gifu University, Gifu 501-1193, Japan; ^2^ Department of General and Gastroenterological Surgery, Osaka Medical College, Takatsuki, Osaka 569-8686, Japan; ^3^ Department of Gastroenterology, Fujita Health University, School of Medicine, Kutsukake-cho, Toyoake, Aichi, 470-1192, Japan; ^4^ Department of Anatomy and Cell Biology, Division of Life Sciences, Osaka Medical College, Takatsuki, Osaka 569-8686, Japan; ^5^ Department of Oncological Surgery, Gifu University School of Medicine, Gifu 501-1193, Japan

**Keywords:** miR-1, miR-133, Warburg effect, PTBP1, PKM

## Abstract

It is known that pyruvate kinase in muscle (PKM), which is a rate-limiting glycolytic enzyme, has essential roles in the Warburg effect and that expression of cancer-dominant PKM2 is increased by polypyrimidine tract-binding protein 1 (*PTBP1*), which is a splicer of the PKM gene. In other words, PKM2 acts as a promoter of the Warburg effect. Previously, we demonstrated that the Warburg effect was partially established by down-regulation of several microRNAs (miRs) that bind to PTBP1 and that ectopic expression of these miRs suppressed the Warburg effect. In this study, we investigated the functions of miR-1 and -133b, which are well known as muscle-specific miRs, from the viewpoint of the Warburg effect in colorectal tumors. The expression levels of miR-1 and -133b were relatively high in colon tissue except muscle and very frequently down-regulated in 75 clinical colorectal tumors samples, even in adenomas, compared with those of the adjacent normal tissue samples. The ectopic expression of these miRs induced growth suppression and autophagic cell death through the switching of PKM isoform expression from PKM2 to PKM1 by silencing PTBP1 expression both *in vitro* and *in vivo*. Also, we showed that the resultant increase in the intracellular level of reactive oxygen species (ROS) was involved in this mechanism. Furthermore, PTBP1 was highly expressed in most of the 30 clinical colorectal tumor samples examined, even in adenomas. Our results suggested that PTBP1 and PTBP1-associated miR-1 and -133b are crucial molecules for the maintenance of the Warburg effect in colorectal tumors.

## INTRODUCTION

The Warburg effect is a well-known feature of cancer cell metabolism [1]. Also, it is well known that there are many factors that establish the Warburg effect, e.g., HIF-1 and c-Myc [2]. Pyruvate kinase in muscle (PKM) is one of the essential molecules for the establishment of the Warburg effect. PKM has 2 isoforms, PKM1 and PKM2. PKM1 contains exon 9 and lacks exon 10. In contrast, PKM2 contains exon 10 and lacks exon 9 [3]. The dominant expression of PKM2 is important for maintenance of the Warburg effect [4]. PKM isoform expression is regulated by the protein family of splicers of heterogeneous nuclear ribonucleoproteins (hnRNPs) [5]. Polypyrimidine tract-binding protein 1 (PTBP1, also known as hnRNPI), is a member of the hnRNP family and promotes PKM2 expression in cancer cells [6]. PTBP1 represses the inclusion of downstream exon by binding to the sequence UCUUC, which exists near the 3′ splice site [7, 8]. This favorable sequence for PTBP1 exists only in intron 8 of PKM mRNA. Hence, PTBP1 represses the inclusion of exon 9 and promotes the expression of PKM2 [6, 9].

MicroRNAs (miRNAs) are single-stranded non-coding small RNAs. They repress gene expression at the translational level by binding to specific complementary sites within the 3′ untranslated region (3′UTR) of targeted mRNAs [10]. The dysregulation of many miRs is responsible for the development of various cancers [11]. Previously, we reported that dysregulation of certain miRs contributes to the development of various cancers including colorectal tumors [12–17].

Recently, we have been focusing on functions of the Warburg effect-associated miRs. Especially, we have investigated the effects of miRs that control PTBP1 expression in cancer cells, because we have found that most primary tumors including colon tumors samples show extreme overexpression of PTBP1. We named these miRs *PTB1* or *PTBP1*-associated miRs. Indeed, we reported that brain-specific miR-124 and muscle-specific-133b contribute a great deal to the establishment of the Warburg effect [18]. Also, miR-124 suppresses the Warburg effect through silencing PTBP1 or DEAD-box RNA helicase 6 (DDX6) in colon cancer cells [19, 20]. So far, miR-1 and -133 are well known as muscle-specific miRs involved in the control of muscle development and disease [21]. Also, it was reported that these miRs are associated with the function and reprograming of the myocardium [22, 23]. However, the functions of these muscle-specific miRs in colorectal tumors have remained unclear. In this present study, we examined the effects of miR-1 and -133b on the Warburg effect in colorectal tumors.

## RESULTS

### MiR-1 and -133b were relatively highly expressed in colon tissue and very frequently down-regulated in clinical colorectal tumor samples

**Figure 1:**
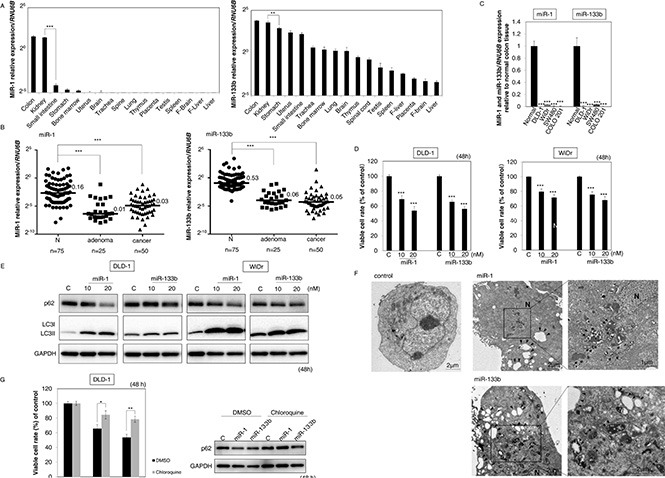
(**A–C**) MiR-1 and -133b very frequently down-regulated in colorectal tumors. (A) Relative expression levels of miR-1 and -133b in various human normal tissues except for skeletal muscle and heart. (B) Dot plot showing the relative expression levels of miR-1 and -133b in tissues of 75 colorectal tumors, consisting of 50 colorectal cancers and 25 colorectal adenomas compared with those of adjacent normal mucosa tissues. The horizontal lines and numbers represent the median values of the distribution. (C) Relative expression levels of miR-1 and -133b in normal colon tissue and in colon cancer cell lines. Colon tissue was obtained from premium total RNA clontech (Takara Bio Company). (**D–G**) MiR-1 and -133b induced autophagic cell death in colon cancer cells. Effects of ectopic expression of miR-1 and -133b on cell viability (D) and expression of LC3I, II, and p62 as estimated by Western blot analysis (E) at 48 h after transfection of DLD-1 and WiDr cells with these miRs at a concentration of 10 or 20 nM. (F) Morphological study using electron microscopy. DLD-1 cells were treated with control miRNA, miR-1 or -133b (20 nM) for 48 h. *N*: nuclei, *Arrowhead:* autophagosome. *Arrow*: mitophagy. The *boxed regions* are shown enlarged beside the main image. (G) Cell viability and expression levels of p62 after combination treatment with chloroquine and both miRs. DLD-1 cells were pretreated with chloroquine (1 μM) at 5 h before the transfection with miR-1 and -133b (20 nM). Results are presented the mean±SD; *P < 0.05; **P < 0.01; ***P < 0.001.

Firstly, we examined the expression profiles of miR-1 and -133b in normal tissues except for muscle. As a result, both miRs were preferentially higher in the tissue from colon than in those from other organs (Figure 1A). Next, we examined the expression levels of both miRs in the tumor samples from the patients with colorectal adenoma or cancer. Surprisingly, down-regulation of both miRs was observed in more than 90% of the samples (Figure 1B and Table 1). It should be noted that the down-regulation of miR-133 occurred more frequently than that of miR-1 (98.7% *vs.* 92.0%) and that the down-regulation was confirmed even in adenoma samples. Also, in the human colon cancer cell lines tested, the expression levels of both miRs were significantly down-regulated (Figure 1C). These findings suggested that dysregulation of the expression of both miR-1 and -133b was closely associated with the development of colorectal tumors.

**Table 1:** Characterictics of study population and expression of miR-1 and -133b in colorectal tumors

Characteristics	*n*	Expression of miR-1 (↓) (cases [%])	Expression of miR-133b (↓) (cases [%])
Total			
Total	75	69 (92)	74 (98.7)
Sex			
Male	52	46 (88.5)	51 (98.1)
Female	23	23 (100)	23 (100)
Tumor			
Cancer	50	46 (92)	50 (100)
Adenoma	25	23 (92)	24 (96)
Location			
Right colon	15	15 (100)	15 (100)
Left colon	60	54 (90)	59 (98.3)
Depth on cancer			
Mucosa (M)	3	3 (100)	3 (100)
Submucosa (SM)	5	5 (100)	5 (100)
Mucosa propria (MP)	10	10 (100)	10 (100)
Subserosa(SS)	20	17 (85)	20 (100)
Serosa exposure, Serosa invasion (SE,SI)	12	11 (91.7)	12 (100)
Tumor diameter in cancer (nm)			
< 47.5	25	23 (92)	25 (100)
> 47.5	25	23 (92)	25 (100)
Dukes classification system			
A	20	19 (95)	20 (100)
B	8	8 (100)	8 (100)
C	20	17 (85)	20 (100)
D	2	2 (100)	2 (100)
Tumor diameter in adenoma (nm)			
< 10	5	5 (100)	4 (80)
≥ 10	20	18 (90)	20 (100)
Grade in adenoma			
Low-grade dysplasia	19	18 (94.7)	18 (94.7)
High-grade dysplasia	6	5 (83.3)	6 (100)

### MiR-1 and -133b induced growth inhibition through autophagic cell death in colon cancer cells

Next, we investigated the effects of these miRs on colon cancer cells, using colon adenocarcinoma cell lines DLD-1 and WiDr. As shown in Figure 1D, the ectopic expression of these miRs induced growth inhibition in both cells. Also, Western blotting analysis showed a significant transition of LC3I to LC3II and decreased expression of p62 (Figure 1E), thus indicating induction of autophagy. Moreover, electron microscopy revealed that the mitochondria of the transfected cells contained many vacuoles, which are a characteristic of autophagy (Figure 1F). Furthermore, the lysosome inhibitor chloroquine increased the number of viable cells in DLD-1 cells transfected with both miRs (Figure 1G). These findings implied that these miRs inhibited growth by triggering autophagic cell death.

### MiR-1 and -133b directly bound to PTBP1

**Figure 2:**
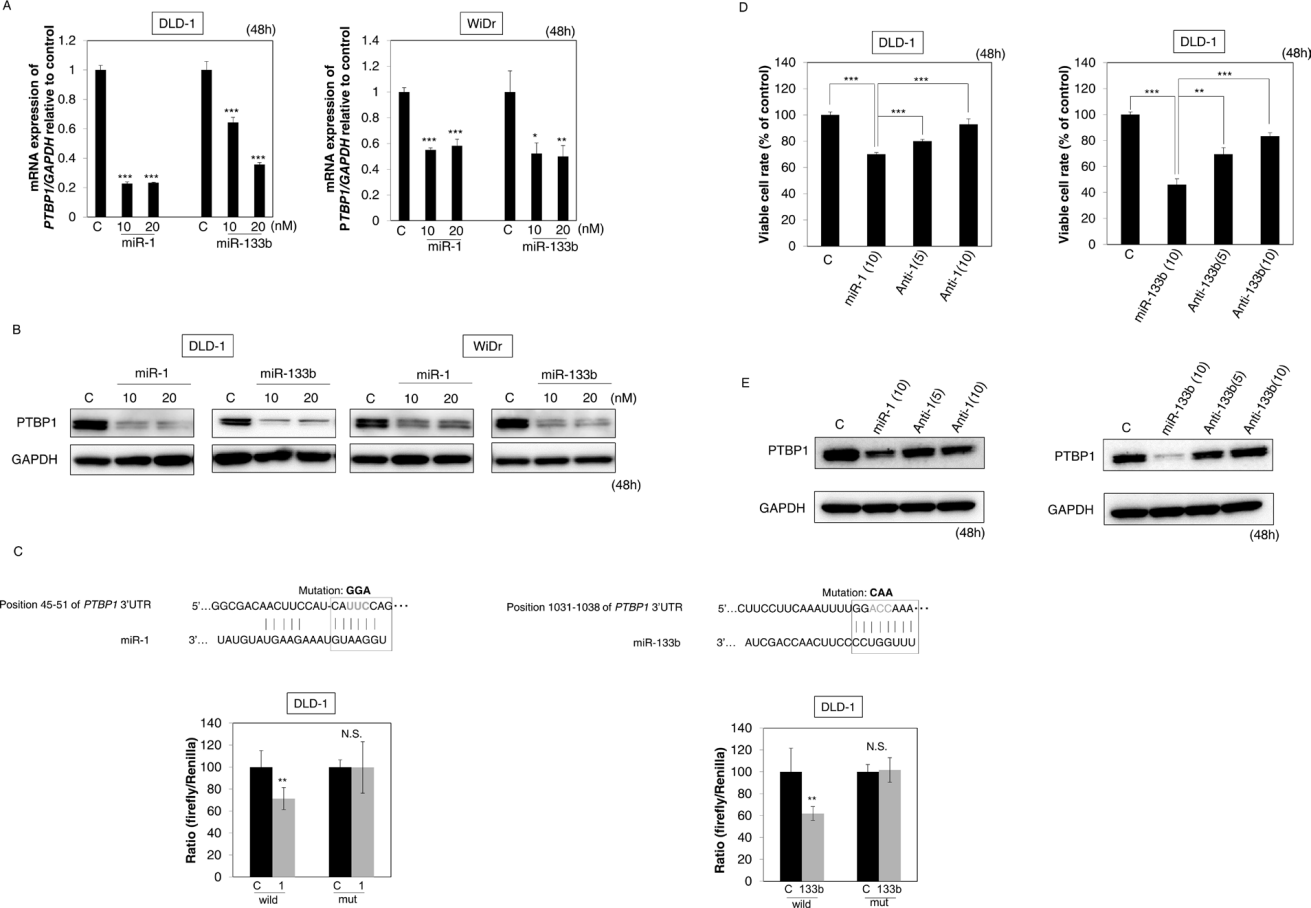
MiR-1 and -133b bind to *PTBP1* (**A, B**) The mRNA expression (A) and protein expression (B) of *PTBP1* at 48 h after the transfection with miR-1 or -133b (10, 20 nM). (**C**) Luciferase activities after co-transfection with control, miR-1 or -133b and wild-type or mutant-type pMIR vectors having the predicted miR-1 or -133b binding site in the 3′UTR of *PTBP1*. The upper panel shows the region of the 3′-UTR of human *PTBP1* mRNA complementary to the mature miR-1 or -133b. The box indicates the predicted binding sites for miR-1 or -133b. (**D**) Effect of combined treatment with antagomiR-1 / miR-1 or antagomiR-133b / miR-133b on the growth of DLD-1 cells. Cells were transfected with non-specific control, miR-1 or -133b (10 nM), miR-1 or -133b (10 nM) + antagomiR-1 or -133b (5 nM) or miR-1 or -133b (10 nM) + antagomiR-1 or -133b (10 nM). (**E**) Expression level of *PTBP1* in DLD-1 cells assessed at 48 h after transfection of the cells. Results are presented as the mean ± SD; *P < 0.05; **P <0.01; ***P < 0.001; N.S., not statistically significant.

Earlier we reported that miR-124 suppresses the Warburg effect via the miR-124/PTBP1/PKMs axis by binding to PTBP1 [19]. The miRNA database indicated that PTBP1 also has predicted binding sites for miR-1 and -133b. Then, to validate whether these miRs indeed bound to PTBP1, we examined the expression levels of PTBP1 after the introduction of either miR into DLD-1 and WiDr cells. As shown in Figure 2, the mRNA (A) and protein (B) levels of PTBP1 were markedly down-regulated in the treated cells. Also, the luciferase reporter activity of wild-type pMIR-PTBP1 was significantly inhibited after the introduction of either miR into DLD-1 cells. On the other hand, mutation of the *PTBP1* 3′-UTR-binding site markedly abolished the inhibitory ability of either miRNA (Figure 2C). Furthermore, treatment with antagomiR-1 or -133b significantly reversed the growth suppression induced by either miR and increased the expression level of *PTBP1* (Figure 2D and 2E). Based on these results, we concluded that miR-1 and -133b directly bound to *PTBP1*.

### MiR-1 and -133b induced switching of PKM isoform expression from PKM2 to PKM1

**Figure 3:**
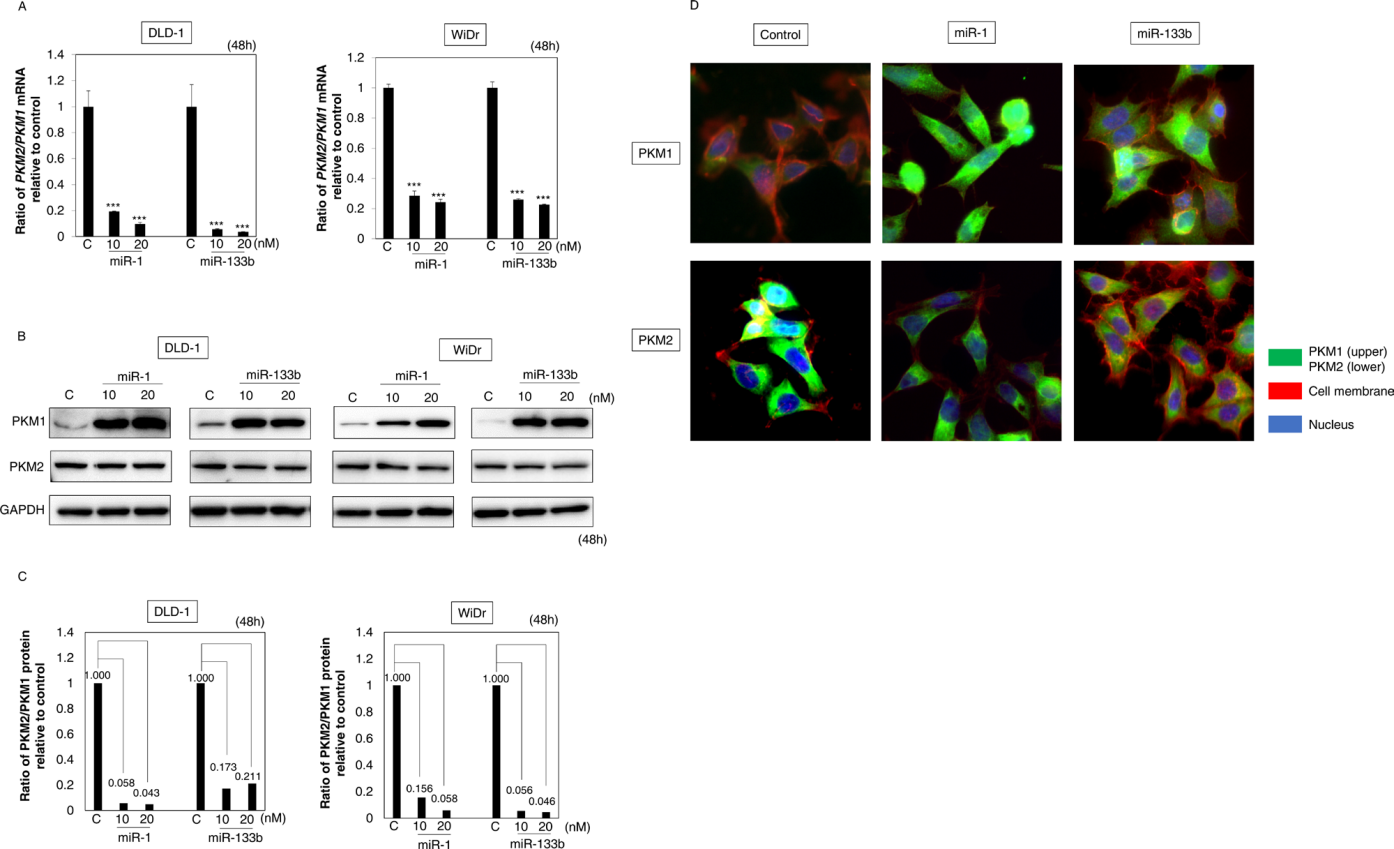
MiR-1 and -133b induced shifting of PKM isoform expression from PKM2 to PKM1 (**A**) mRNA expression of *PKM1* and *PKM2* at 48 h after the transfection of DLD-1 and WiDr cells with miR-1 or -133b (10, 20 nM). The *PKM2/PKM1* ratio was calculated based on their relative mRNA levels. (**B**) Protein expression of *PKM1* and *PKM2* at 48 h after the transfection of DLD-1 and WiDr cells with miR-1 or -133b (10, 20 nM). (**C**) PKM2/PKM1 ratio calculated based on densitometric values of PKM1 and PKM2 in “B”. Numbers represent ratios when control values were taken as 1.000. (**D**) Immunofluorescence of PKM1 (upper panels) and PKM2 (lower panels) at 48 h after transfection of DLD-1 cells with miR-1 or -133b (20 nM). Left panels, treatment with control miRNA; Middle panels, treatment with miR-1, Right panels, treatment with miR-133b. PKM1 or PKM2 is stained green, cell membrane is stained red, and nuclei are stained blue. Results are presented as the mean ± SD; ***P < 0.001.

As mentioned earlier in the Introduction section, PTBP1 is well known as a promoter of PKM2 expression [5, 24]. Indeed, we confirmed presently that overexpression of PTBP1 increased expression of PKM2 (Supplementary Figure S1A). On the other hand, knockdown of PKM2 decreased expression of PTBP1 (Supplementary Figure S1B). Also, we found that miR-1 and -133b bound to PTBP1 (Figure 2). Based on these findings, we hypothesized that both miRs induced switching of the PKM isoform expression from the cancer-dominant PKM2 to PKM1. In fact, the ratio of PKM2/PKM1 mRNAs was significantly decreased after the treatment with either miR (Figure 3A). Also, Western blotting analysis showed that the expression of PKM2 protein was slightly down-regulated and that of PKM1 was significantly up-regulated in all treated cells (Figure 3B and 3C). Furthermore, immunofluorescence (IFC) indicated that immunostaining for PKM1 was markedly increased in intensity, but that that for PKM2 was slightly decreased, in the treated-DLD-1 cells (Figure 3D). Therefore, this switching by these miRs was found even at the single-cell level. These findings suggested that these miRs negatively regulated the cancer-dominant PKM2 expression through the binding to *PTBP1*.

### Ectopic expression of miR-1 and -133b suppressed the Warburg effect

**Figure 4:**
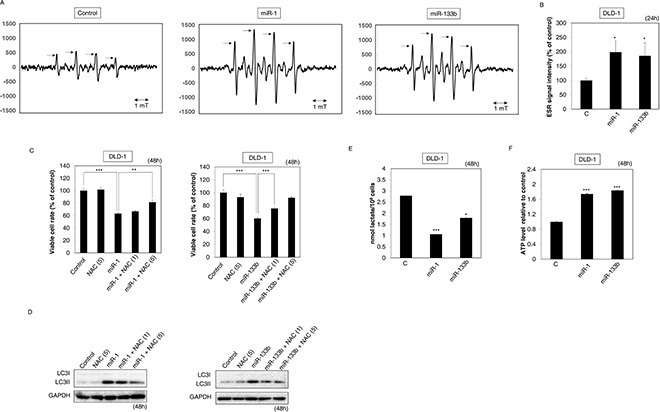
Ectopic expression of miR-1 and -133b suppressed the Warburg effect (**A, B**) Free radicals were evaluated by ESR at 24 h after the transfection of DLD-1 cells with miR-1 or -133b (20 nM). The representative ESR spectra are shown in “A” with the arrows indicating O_2_– (superoxide ion). The intensities of the spin adducts are shown in “B”. (**C, D**) Effects of NAC on the growth of DLD-1 cells after their transfection with miR-1 or -133b. DLD-1 cells were treated with NAC (1, 5 mM) at 24 h after the transfection with miR-1 or -133b (20 nM). (C) Cell viability of combination treatment with NAC and these miRs. (D) Western blot analysis at 48 h after the transfection of DLD-1 cells with NAC and these miRs. (**E**) Lactate production was measured at 48 h after the transfection of DLD-1 cells with miR-1 or -133b (20 nM). (**F**) ATP levels were measured under the same conditions as for lactate measurement. Results are presented as the mean ± SD; *P < 0.05; **P <0.01; ***P < 0.001.

PKM1 is well known as promoter of the TCA cycle; and PKM2, as a promoter of glycolysis [5, 6, 24]. Therefore, we hypothesized that ectopic expression of these miRs would suppress the Warburg effect through this isoform-switching machinery. To examine the validity of this hypothesis, we measured the level of superoxide ion (O_2_−) by ESR after treating DLD-1 cells with either of these miRs. As a result, the levels of O_2_− were significantly increased in the treated-DLD-1 cells (Figure 4A and 4B). Next, we examined whether the effects of these miRs would be canceled by NAC, a free-radical scavenger. NAC significantly inhibited the growth suppression by these miRs (Figure 4C). Also, Western blotting analysis indicated that the transition of LC3I to II induced by these miRs was reduced by the treatment in the presence of NAC (Figure 4D). Next, we examined lactate production, the final product of glycolysis, after the treatment with either miR. Expectedly, this production was significantly decreased in DLD-1 cells treated with either miR (Figure 4E). Furthermore, ATP levels were significantly increased in DLD-1 cells transfected with either miR (Figure 4F). These findings suggested that the ectopic expression of these miRs suppressed growth by impairing the Warburg effect in colon cancer cells.

### Knockdown of PTBP1 had the same effects as those induced by the ectopic expression of these miRs in colon cancer cells

**Figure 5:**
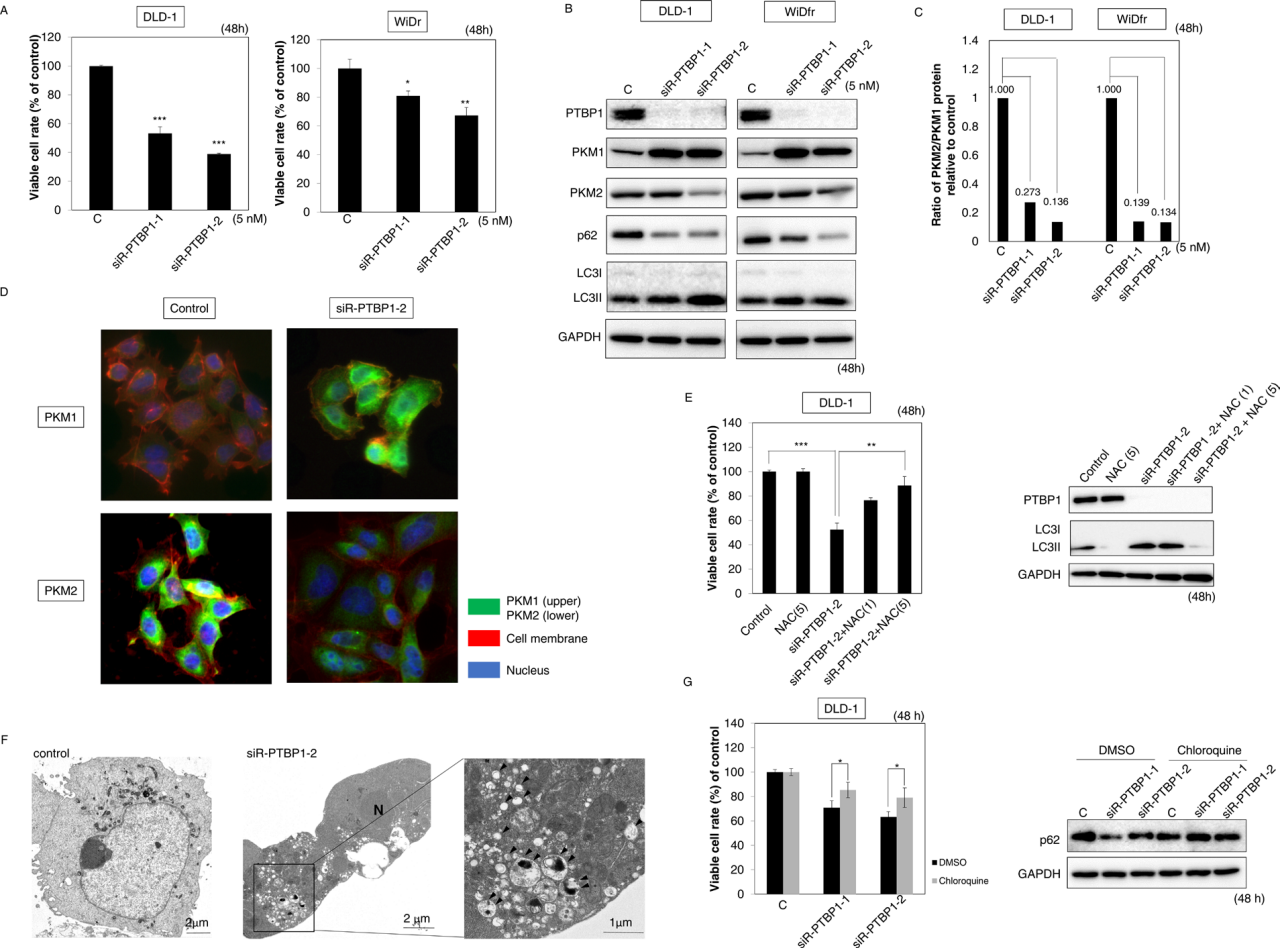
Knockdown of *PTBP1* induced the same effects as did miR-1 and -133b (**A**) Effects of *PTBP1* knockdown on cell growth. (**B**) Various protein expression in DLD-1 and WiDr cells, as examined by Western blot analysis at 48 h after siR-PTBP1 transfection at a concentration of 5 nM. (**C**) The PKM2/PKM1 ratio of was calculated based on densitometric value of PKM1 and PKM2 in “B”. Numbers represent PKM2/PKM1 ratio of each sample, with the control taken as 1.000. (**D**) Immunofluorescence of PKM1 (upper panels) and PKM2 (lower panels) at 48 h after transfection of DLD-1 cells with siR-PTBP1-2 (3′-UTR, 5 nM). Left panels, treatment with control miRNA; Right panels, treatment with siR-PTBP1-2. PKM1 or PKM2 is stained green, cell membrane is stained red, and nuclei are stained blue. (**E**) Effects of NAC on the growth of DLD-1 cells after their transfection with siR-PTBP1-2. DLD-1 cells were treated with NAC (1, 5 mM) at 24 h after the transfection with siR-PTBP1-2 (5 nM). Viability of cells after combination treatment with NAC and siR-PTBP1-2 (Left) and results of Western blot analysis (Right) at 48 h after the combination treatment are shown. (**F**) Morphological study using electron microscopy. DLD-1 cells were treated with control miRNA or siR-PTBP1-2 (5 nM) for 48 h. *N*: nuclei, *Arrowhead*: autophagosome. The *boxed regions* are shown enlarged beside the main image. (**G**) Cell viability and expression levels of p62 after combination treatment with chloroquine and siR-PTBP1. DLD-1 cells were pretreatment with chloroquine (1 μM) at 5 h before the transfection with siR-PTBP1 (5 nM). Results are presented as the mean±SD; *P < 0.05; **P < 0.01; ***P < 0.001.

To clarify whether these anti-cancer effects induced by miR-1 and -133b depended on the binding to *PTBP1*, we examined the effects of knockdown of *PTBP1* on colon cancer cells. We were able to silence *PTBP1* by binding siRNA to its ORF (siR-PTBP1-1) or 3′UTR (siR-PTBP1-2). As a result, both types of siR-PTBP1 induced a significantly inhibited the growth of either DLD-1 or WiDr cells (Figure 5A). The growth-suppressive effect of siR-PTBP1-2 was greater than that of siR-PTBP1-1. In addition, Western blotting analysis showed that the switching from PKM2 to PKM1, the transition of LC3I to LC3II, and the decrease in p62 expression had occurred in the siR-PTBP1-transfected cells (Figure 5B and 5C). Also, IHC showed the clear switching from PKM2 to PKM1 at the single-cell level (Figure 5D). Moreover, this growth suppression with autophagy was canceled by NAC (Figure 5E). Electron microscopy showed that many autophagosomes were present in siR-PTBP1-treated cells as in the case of miR-1 or -133b-treated cells (Figure 5F). Furthermore, chloroquine recovered the viability of siR-PTBP1-transfected DLD-1 cells (Figure 5G). The decrease in lactate production by knockdown of PTBP1 was already reported by us and others [5, 6, 19]. These findings suggested that the miR-1 and -133b/PTBP1 axis was essential for the maintenance of the Warburg effect in colon cancer cells.

### Anti-tumor effect of miR-1and -133b on xenografted tumor in nude mice

**Figure 6:**
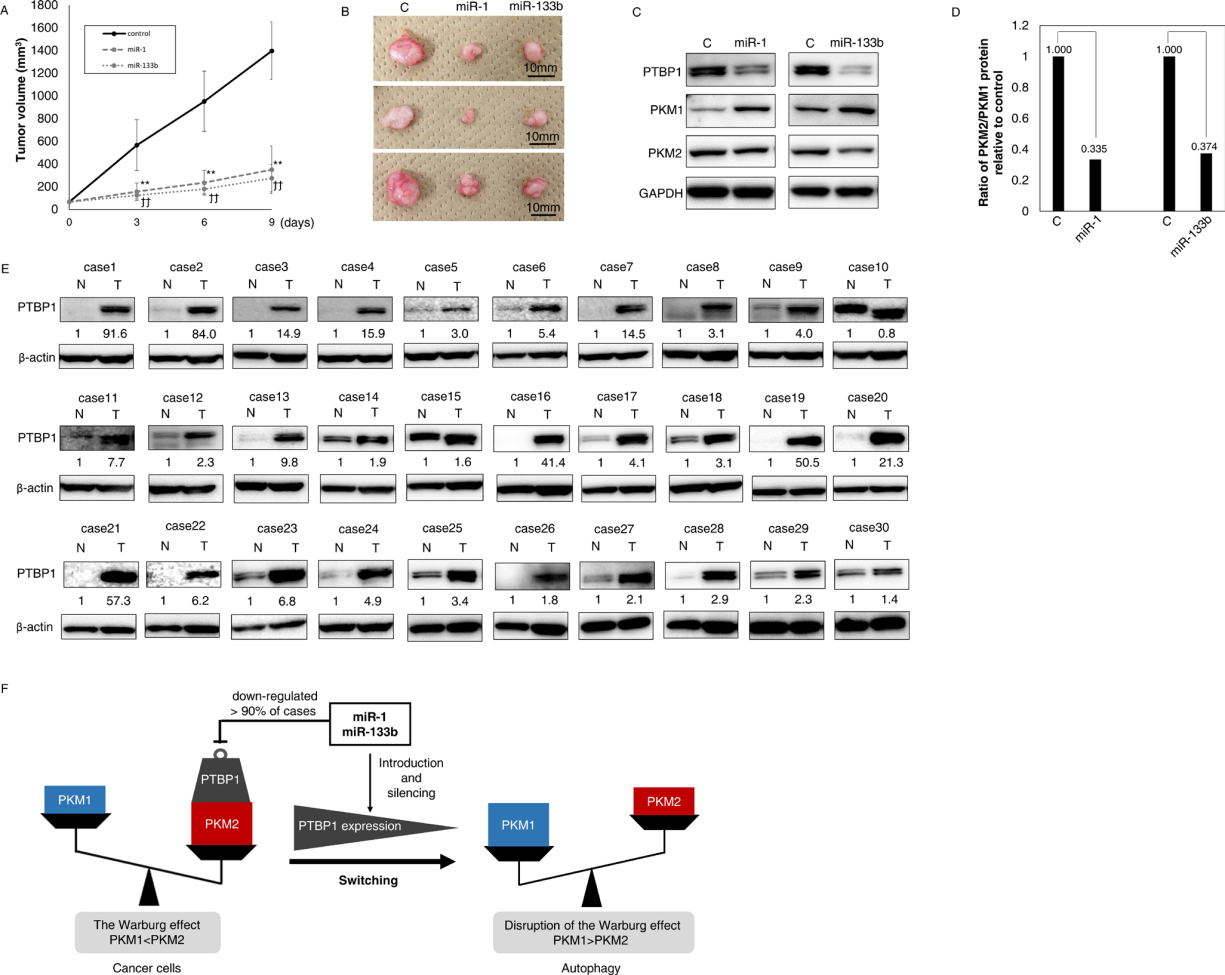
(**A**–**D**) MiR-1 and -133b have anti-tumor effects even *in vivo*. (A) Time course of tumor size in mice injected with control miRNA, miR-1 or -133b (*n* = 5). (B) Representative photograph of tumors. Left is the control. Middle is treatment with miR-1; and right, treatment with miR-133b. (C) PTBP1 and PTBP1-related protein expression in control, miR-1or -133b-treated tumor tissues as determined by Western blot analysis. (D) The ratio of PKM2/PKM1 protein was calculated based on densitometric value of PKM1 and PKM2 in “C”. Numbers represent the PKM2/PKM1 ratio of each sample, with the control taken as 1.000. (**E**) PTBP1 was overexpressed in clinical colorectal tumors. PTBP1 expression in 25 colorectal cancer (case 1–25) and 5 adenoma (case 26–30) samples as determined by Western blot analysis. Details of the characteristics of the samples are given in Supplementary Table 1. Densitometric values of PTBP1 were calculated. β-actin was used as the control. (**F**) Schematic diagram of the effects of miR-1 and -133b on colorectal tumors. MiR-1 and -133b directly bind to *PTBP1*, and *PTBP1* is very frequently overexpressed in colorectal tumors and even in adenomas, with the overexpression induced by down-regulation of these miRs. Ectopic expression of these miRs induced switching of PKM isoform expression from PKM2 to PKM1 through down-regulation of PTBP1. These miRs induce autophagic cell death and the activation of oxidative stress through this pathway. These miRs and PTBP1 have great potential as therapeutic targets or as biomarkers in colorectal tumors.

To examine the anti-tumor effect of these miRs *in vivo*, we inoculated DLD-1 cells subcutaneously into nude mice. After confirmation of tumor engraftment, we injected control miR, miR-1 or -133b into the developed tumor. As shown in Figure 6A and 6B, significant growth suppression of tumors was observed in the group treated with miR-1 or -133b. Western blotting analysis of samples of grafted tumors showed that the switching of PKM isoform expression from PKM2 to PKM1 through down-regulation of PTBP1 had occurred, as had been found *in vitro* (Figure 6C and 6D). These findings suggested that both miRs induced growth inhibition of the engrafted tumor through down-regulation of *PTBP1* even *in vivo*.

### PTBP1 was over-expressed in clinical colorectal tumor samples

Finally, to investigate whether PTBP1 functioned as an oncogene clinically, we examined the expression level of PTBP1 in clinical colorectal tumor samples. We examined the expression level of PTBP1 in 30 cases (25 cancer and 5 adenoma samples), which were also used for Western blotting analysis (Supplementary Table S1). As shown in Figure 6E, PTBP1 expression was significantly higher in all cancer samples except for case 10. Interestingly, PTBP1 expression was already up-regulated in all adenoma samples tested (Figure 6E). These findings strongly suggested that PTBP1 was oncogenic, working at the initiation of the adenoma-carcinoma sequence triggered in part by the down-regulation of *PTBP1-*associated miR-1 and -133b (Figure 6F).

## DISCUSSION

In this study, we showed that miR-1 and -133b were relatively highly expressed in colorectal tissue and very frequently (more than 90%) down-regulated during tumor development, even at the adenoma stage (Figure 1A, 1B and Table 1). The dysregulation of these miRs contributed to carcinogenesis in the early phase of the adenoma-carcinoma sequence. It has been reported that epigenetic alterations are a representative mechanism for the dysregulation of miRs [25, 26]. Therefore, we examined the effect of demethylation by 5-Aza on various colon cancer cells. As expected, 5-Aza treatment significantly increased the expression levels of miR-1 and -133b and down-regulated PTBP1 in almost all cell lines tested (Supplementary Figure S2A and S2B). These results implied that hypermethylation of these miRs was one of the reasons for their dysregulation. Further investigation is needed to clarify the mechanism of down-regulation of such muscle-specific miRs during colorectal tumor development.

Notably, we showed that the introduction of these miRs induced metabolic switching from glycolysis to TCA cycle along with oxidative phosphorylation to produce ATP (Figure 4E and F). On the other hand, Figure 4A, 4C, and 4D showed that generation of ROS was correlated with the autophagic cell death. Based on these results, we presumed that intracellular ROS was increased drastically by the abrupt metabolic change and that finally autophagic cell death resulted from impairment of cellular organelles, especially mitochondria, and metabolic pathways. In fact, the addition of neither Glutamine nor fatty acid could recover the viability of cells transfected with either miR (Supplementary Figure S3A). Also, the expression level of mTOR, which is a representative negative regulator of autophagy, was decreased in both miRs- and siR-PTBP1-transfected cells (Supplementary Figure S3B and S3C).

Another important conclusion of this study is that PTBP1 is a very crucial oncogene in the development of colorectal tumors. So far, many functions of PTBP1 have been reported, especially regarding mRNA metabolism. PTBP1 was originally cloned as an essential molecule in the alternative splicing of α-tropomyosin [27] and is known as a negative regulator of alternative splicing by exon repression in various pre-mRNAs [7, 8]. PTBP1 has been implicated in the polyadenylation of the pre-mRNA 3′end [28]. Also, PTBP1 increases mRNA stability by binding to the 3′-UTR of mRNAs such as those for rat insulin and vascular endothelial growth factor (VEGF) [29–31]. Moreover, PTBP1 exports mRNA from the nucleus and localizes it in the cytoplasm [32, 33]. Another interesting function of PTBP1 is its promotion of cap-independent translation through mediation of the internal ribosomal entry site (IRES) [34, 35]. In cancer, it has been reported that PTBP1 is over-expressed in glioblastoma, ovarian tumor tissues, and breast cancer cells [36–38]. In this study, we indicated that PTBP1 was very frequently over-expressed in colorectal tumors and even in adenomas. This finding supports the notion that colorectal adenoma has already acquired the machinery needed for the Warburg effect. Moreover, in Figure 6E, the samples of case 25 and case 30 were acquired from the same patient. Case 25 was an early adenocarcinoma; and case 30, a high-grade adenoma. The PTBP1 expression was more up-regulated in the adenocarcinoma than in the high-grade adenoma when each expression was compared with that in the normal tissue (Figure 6E). These results implied that PTBP1 expression was further up-regulated in the process from adenoma to adenocarcinoma and affected the malignant process in carcinogenesis.

On the other hand, c-Myc was reported as a positive regulator of PTBP1 [6]. However, the regulatory mechanism of PTBP1 has largely remained unclear. Recently, in elucidating the PTBP1 regulatory mechanism from the point of view of miRs, we succeeded in showing that PTBP1 is negatively regulated by organ-specific miRs such as miR-124 [18, 19]. *PTBP1*-associated miRs such as miR-1, -9, -124, -133, and -137 are distributed in high energy-demanding organs such as brain, skeletal muscle, and heart. Accordingly, PKM1 is dominantly expressed in only these organs; because these organs need efficient acquisition of energy from glucose [18]. We consider that these organs make PKM1 dominant through the appropriate distribution of *PTBP1*-associated miRs *in vivo*. Furthermore, PTBP1 has a negative role in the differentiation of skeletal muscle or neural cells by alternative splicing, as described above [8]. It has been reported that a part of the differentiation machinery in neural cells is controlled by PTBP1-associated miR-124 [39]. Therefore, we speculate that these miRs are fine tuners to regulate PTBP1 expression *in vivo* and that the dysregulation of these miRs induces the overexpression of PTBP1, leading to the establishment of the malignant phenotype in cancer cells such as the Warburg effect. It should be clarified how PTBP1 is dysregulated especially in the process of carcinogenesis.

Now, there is a trend for the use of tyrosine kinase inhibitors in clinical medicine. However, in many cases the survival of patients can be prolonged only for a short period of time. One of the main reasons for this short survival is reprograming of the tumor environment by activated compensatory cascades. However, the results of the present study strongly suggest that *PTBP1*-associated miRs and *PTBP1* have the great potential of being target molecules for therapy or being biomarkers, because the mechanisms at play are essential for cancer cells during malignant transformation; and, furthermore, they can affect the entire cancer-specific energy metabolism. Now, we are seeking to elucidate the functions of *PTBP1*-associated miRs and *PTBP1* in many other kinds of cancer cells. We expect further progress in elucidation of the detailed networks of Warburg effect-associated molecules in the near future.

## MATERIALS AND METHODS

### Patients and samples

All human samples were obtained from patients who had undergone biopsy or surgery for resection at Fujita Health University Hospital (Toyoake, Aichi, Japan), Gifu University Hospital (Gifu, Gifu, Japan), Misao Health Clinics (Gifu, Gifu, Japan), Osaka Medical College Hospital (Takatsuki, Osaka, Japan) or Saiseikai Ibaraki Hospital (Ibaraki, Osaka, Japan). Collection and distribution of the samples were approved by each of the appropriate institutional review boards in accordance with the Declaration of Helsinki. Fifty patients with previously untreated (or recently diagnosed) colorectal cancer and 25 with adenomas were selected. The distribution according to other clinical parameters is shown in Table 1. Under a pathologist's supervision, all tissue sample pairs were collected from surgically or endoscopically resected tissues, with these paired samples being from the primary tumor and its adjacent non-tumor mucosal tissue in the same patient.

### Cell culture and cell viability

All cell lines were obtained from JCRB (Japanese Collection of Research Bioresources) Cell Bank. All cell lines were cultured in RPMI-1640 medium supplemented with 8% (v/v) heat-inactivated FBS (Sigma-Aldrich Co, St. Louis, MO USA) and 2 mM L-glutamine under an atmosphere of 95% air and 5% CO_2_ at 37°C. The number of viable cells was determined by performing the trypan-blue dye exclusion test.

### Transfection experiments

DLD-1 cells or WiDr cells were seeded in 6-well plates at a concentration of 0.5 × 10^5^ per well (10–30% confluence) on the day before the transfection. The mature types of miR-1 and-133b (mirVana^™^ miRNA mimic; Ambion, Foster City, CA, USA), antagomiR-1 and -133b (mirVana^™^ miRNA inhibitor; Ambion) or siRNA for *PTBP1* (siR-PTBP1; Invitrogen Carlsbad, CA) was used for the transfection of the cells, which was achieved by using cationic liposomes, Lipofectamine^™^ RNAiMAX (Invitrogen), according to the manufacturer's Lipofection protocol. The nonspecific miRNA (HSS, Hokkaido, Japan) sequence of 5′-GUAGGAGUAGUGAAAGGCC-3′ was used as a control for nonspecific effects [17]. The sequence of the mature type of miR-1 used in this study was 5′-UGGAAUGUAAAGAAGUAUGUAU-3′; that of miR-133b, 5′-UUUGGUCCCCUUCAACCAGCUA-3′; that of siR-*PTBP1* for the open reading frame region, 5′-UGUCAUUUCCGUUUGCUGCAGAAGC-3′; and that of the 3′UTR region, 5′-AUCUCUGGUCUGCUAAGG UCACUUC-3′. The effects manifested by the introduction of miRs and siRNAs into the cells were assessed at 48 h after the transfection.

### Inhibitor agents

We used the lysosome inhibitor chloroquine and free-radical scavenger N-acetyl-L-cysteine (NAC), both obtained from Sigma Aldrich (St Louis, MO, USA). DLD-1 cells were treated with chloroquine (1 μM) 5 h before transfection with miR-1, -133b or siR-PTB1. Also, DLD-1 cells were treated with NAC (1, 5 mM) 24 h after transfection with miR-1, -133b or siR-PTB1.

### Western blotting

Whole cells were homogenized in chilled lysis buffer comprising 10 mM Tris-HCl (pH 7.4), 1% NP-40, 0.1% deoxycholic acid, 0.1% SDS, 150 mM NaCl, 1 mM EDTA, and 1% Protease Inhibitor Cocktail (Sigma-Aldrich Co.) and stood for 20 min on ice. After centrifugation at 13,000 rpm for 20 min at 4°C, the supernatants were collected as whole-cell protein samples. Protein contents were measured with a DC Protein assay kit (Biorad, Hercules, CA, USA). Ten micrograms of lysate protein was separated by SDS-PAGE using 10.0 or 12.5% polyacrylamide gels, and electroblotted onto a PVDF membrane (PerkinElmer Life Sciences, Inc., Boston, MA, USA). After blockage of nonspecific binding sites for 1 h with 5% nonfat milk in PBS containing 0.1% Tween 20 (TBS-T), the membrane was incubated overnight at 4°C with primary antibodies. The next day, the membrane was then washed 3 times with TBS-T, incubated further with secondary antibodies at room temperature for 1 hour, and then washed 3 times with TBS-T. The immunoblots were visualized by use of Ammersham ECL Plus Western Blotting Detection Reagents (GE Healthcare, Buckinghamshire, UK). Primary antibodies against the following antigens were used: PTBP1, LC3B, p62, GAPDH (Cell Signaling Technology, Inc., Danvers, MA, USA); PKM1 and PKM2 (Novus Biologicals, USA); and β-actin (Sigma-Aldrich Co.). HRP-conjugated goat anti-rabbit and horse anti-mouse IgG (Cell Signaling Technology) were used as secondary antibodies. GAPDH or β-actin was used as an internal control.

### Real-time reverse transcription-PCR

Total RNA was isolated from cultured cells or tumor tissues by using a NucleoSpin microRNA isolation kit (TaKaRa, Otsu, Japan) or Trizol reagent (Invitrogen) followed by DNase I treatment. RNA concentrations and purity were assessed by UV spectrophotometry. The primers for *PTBP1, PKM1, PKM2, and GAPDH* were the following:*PTBP1*-sense, 5′-ATC AGG CCT TCA TCG AGA TGC ACA-3′, and *PTBP1*-antisense, 5′-TGT CTT GAG CTC CTT GTG GTT GGA-3′; *PKM1*-sense, 5′-CGA GCC TCA AGT CAC TCC AC-3′, and *PKM1*-antisense, 5′-GTG AGC AGA CCT GCC AGA CT-3′; *PKM2*-sense, 5′-ATT ATT TGA GGA ACT CCG CCG CCT-3′, and *PKM2*-antisense, 5′-ATT CCG GGT CAC AGC AAT GAT GG-3′; *GAPDH*-sense, 5′-CCA CCC ATG GCA AAT TCC ATG GCA-3′, and *GAPDH*-antisense, 5′-TCT AGA CGG CAG GTC AGG TCC ACC-3′. *RNU6B* and *GAPDH* were used as an internal control. The relative expression levels were calculated by the ΔΔCt method.

### Luciferase reporter assay

Searching the Target Scan 6.2 database (http://www.targetscan.org/) to find algorithm-based binding sites of miR-1 or -133b, we found the predicted binding site to be at position 45-51 for miR-1 and at 1031-1038 for miR-133b in the 3′UTR of *PTBP1* mRNA. The sequence region containing the putative binding sequence of miR-1 or -133b was inserted into a pMIR-REPORT^™^ Luciferase miRNA Expression Reporter Vector (Applied Biosystems) according to the manufacturer's protocol. Moreover, we made other pMIR constructs, one encompassing a mutated seed sequence for miR-1 (wild type, CATTCCA; mutant, CAGGACA) and the other, for -133b (wild type, GGACCAAA; mutant, GGCAAAAA) by using a PrimeSTAR^®^ Mutagenesis Basal Kit (TaKaRa). The mutation of the vector was confirmed by sequence analysis. pRL-TK *Renilla* Luciferase Reporter vector (Promega, Madison WI, USA) was used as an internal control vector. DLD-1 were seeded into 96-well plates at a concentration of 0.1 × 10^4^ per well on the day before the transfection. DLD-1 cells were co-transfected with either reporter vector (0.01 μg/well each) and 20 nM miR-1,-133b or nonspecific non-coding siRNA (Dharmacon, Tokyo, Japan). Luciferase activities were measured at 24 h after co-transfection by using a Dual-Glo Luciferase Assay System (Promega) according to the manufacturer's protocol. Luciferase activities were reported as the firefly luciferase/*Renilla* luciferase ratio.

### Electron microscopic study

We transfected DLD-1 cells with nonspecific control miRNA, miR-1, -133b (20 nM) or siR-PTBP1 (5 nM). At 48 h after the transfection, the effects were manifested. The details of experiment were described in previous our reports [19, 40].

### Immunofluorescence study

DLD-1 cells were seeded into the wells of a Lab-Tek II Chamber Slide System (Thermo Fisher Scientific Inc., Waltham, MA), each well containing 1.0 ml of culture medium plus 10% (w/v) fetal bovine serum. We transfected DLD-1 cells with nonspecific control miRNA, miR-1, -133b (20 nM) or siR-PTBP1 (5 nM). After 48 h of treatment, the cells were immunostained with anti-PKM1 or PKM2 antibody according to the immunofluorescence protocol of Cell Signaling Technology. The nuclei were stained with Hoechet33342, and for actin labeling the cells were incubated with the fluorescent F-actin probe Rhodamine Phalloidin (Cytoskeleton, Denver, CO). The cells were observed with a BIOREVO fluorescence microscope (Keyence, Osaka, Japan).

### Electron spin resonance spectroscopy (ESR)

The production of free radicals was determined by using the ESR trapping technique in combination with 5, 5-dimethyl-1-pyrroline-*N*-oxide (DMPO; Tokyo Chemical Industry Co.,). ESR spectra of free radicals were obtained by using a quartz capillary tube (i. d. 0.75 mm, JES-LC01) and a JOEL JES-FR30EX free-radical monitor (JOEL Ltd., Akishima, Japan). The measurement conditions were the following: magnetic field, 336 ± 5 mT; power, 8.98 mW; sweep time, 4 min; modulation, 100 kHz; width, 0.08 mT; amplitude, 2000; time constant, 0.3 s. DLD-1 cells were treated with control miRNA, miR-1 or -133b (20 nM). Signal intensity was compared between the control and miRs at 24 h after the transfection.

### Lactate assay

DLD-1 cells were collected at 48 h after the transfection with miRs. Intracellular lactate was measured with an L-Lactate Assay kit according to the manufacturer's instructions (Cayman Chemical Company, Ann Arbor, MI, USA). Lactate production was normalized to the number of cells.

### ATP assay

DLD-1 cells were collected at 48 h after the transfection with miRs. ATP production was measured with an ATP Determination Kit according to the manufacturer's instructions (Invitrogen). ATP production was normalized to cell numbers.

### Human tumor xenograft model

Animal experimental protocols were approved by the Committee for Animal Research and Welfare of Gifu University. BALB/cSLC-nu/nu (nude) mice were obtained from Japan SLC (Hamamatsu, Japan). Human colon cancer DLD-1 cells were inoculated at 4.0 × 10^6^ cells/100 μl per site into the back of each mouse. At 7–10 days after the inoculation, we confirmed the engraftment of the tumors. When the tumor size had reached 5 × 5 mm, the treatment was started; and this day was set as day 0. After miR-1, -133b or control miRNA (0.2 nmol per 1 administration) in 100 μl of Opti-MEM had been incubated with 1 μl of Lipofectamine RNAiMAX, the mixture was injected into the tumor every 3 days for a total of 3 times. Each group contained 5 mice. The tumor volume was calculated by the formula: 0.5236 L_1_ (L_2_)^2^, where L_1_ is the long axis and L_2_ is the short axis of the tumor. This formula was described in a previous report [19, 41].

### Statistics

Each examination was performed in triplicate. In experiments on clinical samples, the expression levels of > 1.5 were designated as up-regulation and those of < 0.67 as down-regulation, which fold changes were obtained from the results of linear discriminant analysis of the miRNA expression patterns from many of our previous reports [13, 16]. For determination of statistical differences between levels of miRs in clinical samples and *in vivo* experiments the data were compared by using the Mann–Whitney *U*-test. For *in vitro* experiments, statistical significances of differences were evaluated by performing the two-sided Student's *t*-test. Also, a part of statistical analyses were performed by using GraphPad Prism software system (GraphPad Software, Inc., La Jolla, CA). The values were presented as the mean ± standard deviation. A *P* value < 0.05 was considered to be statistically significant.

## SUPPLEMENTARY MATERIALS FIGURES AND TABLE


