# Supplementary material for: PTBP1-associated microRNA-1 and -133b suppress the Warburg effect in colorectal tumors
Source: Oncotarget. 2016 Mar 9;7(14):18940–52. doi: 10.18632/oncotarget.8005 (PMC4951342; doi:10.18632/oncotarget.8005)
Supplement: Supplementary file 1 [file oncotarget-07-18940-s001.pdf]

PTBP1-associated microRNA-1 and -133b surpress the Warburg effect in colorectal tumors

Supplementary Materials

MATERIALS AND METHODS

Western blotting analysis

Primary antibodies used were as follow: anti-mTOR (Cell Signaling Technology, Inc., Danvers, MA, USA). Information on other primary antibodies were described in the main text.

Gene silencing

The sequence of siR-PKM2 was 5′-CAGAC UUGGUGAGGACGAUUAUGGC-3′.

PTBP1 overexpression experiments

The PTBP1 expression vector was constructed by insertion of the open reading frame (ORF) of PTBP1 cDNA into the SgfI and PmeI sites of the pF5A-CMV *neo* vector (Promega). DLD-1 was used as template of PTBP1 cDNA. Human PTBP1 was cloned by polymerase chain reaction (PCR) amplification from human brain cDNA (QUICK-CloneTM cDNA, Clontech). The primers for ORF-PTBP1 were the following: ORF-PTBP1-sense, 5′-GGGCGATCGCCATGGACGGCATTGTCCCAG-3′, and ORF-PTBP1-antisense, 5′-CCGTTTAAACCTAGAT GGTGGACTTGGAGA-3′. Amplified fragments were digested by Flexi enzyme mix (Promega) and ligated into the pF5A-Flag-neo vector. On the day before the transfection, the cells were seeded into 6-well plates at a concentration of 0.5×10^5/well. We transfected the cells at 0.1 µg/well with the control vector or pF5A-PTBP1 expression vector by using Lipofectamine 2000 (Invitrogen). The effects were assessed at 24 h after the transfection.

Agents

5-Aza-2′-deoxycytidine (5-Aza) was obtained from Sigma Aldrich (St Louis, MO, USA). All cells tested were treated with 5-AZA (5, 10 µM). The effects of 5-Aza were assessed at the appropriate time for each cell line. The lipid mixture used was obtained from Sigma-Aldrich (St Louis, MO, USA). It contained arachidonic acid, linoleic acid, linolenic acid, myristic acid, palmitic acid, and stearic acid and was designated as FA. We added FA at 10 µl/1 ml of medium. L-Glutamine was obtained from Thermo Fisher Scientific (Waltham, MA USA). We added 200 mM L-Glutamine at 10 µl /1 ml of medium.

Detail methods of experiments were described in main text.

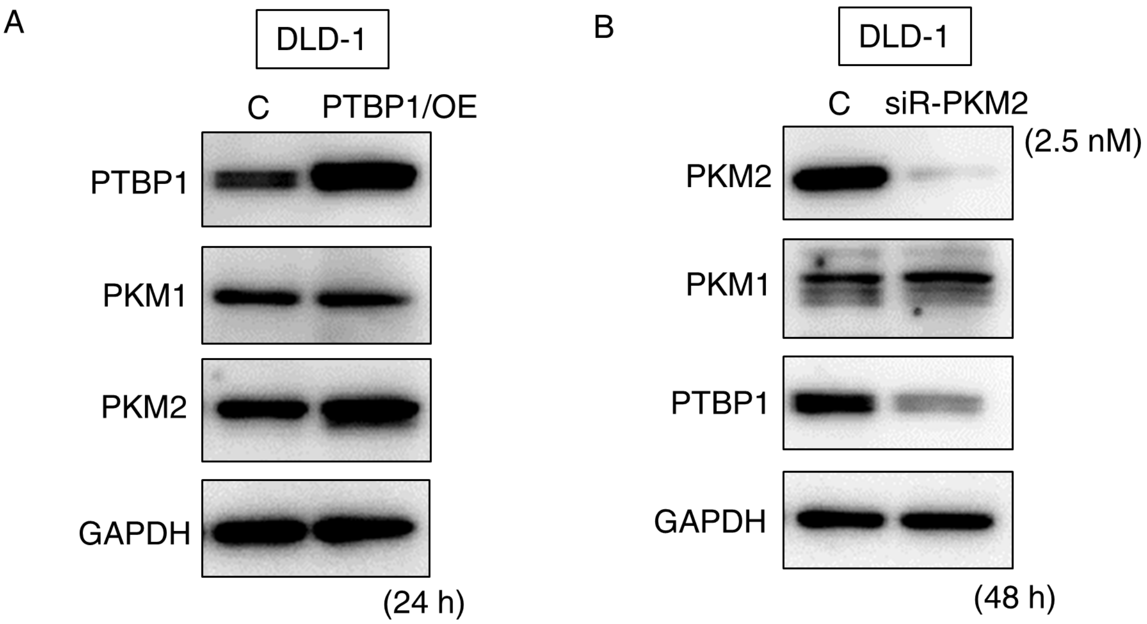

Supplementary Figure S1: (**A**) Protein expression of Warburg effect-associated genes at 24 h after transfection of DLD-1 cells with pF5A-PTBP1 (0.1 µg). (**B**) Protein expression of Warburg effect-associated genes at 48 h after transfection of DLD-1 cells with siR-PKM2 (2.5 nM).

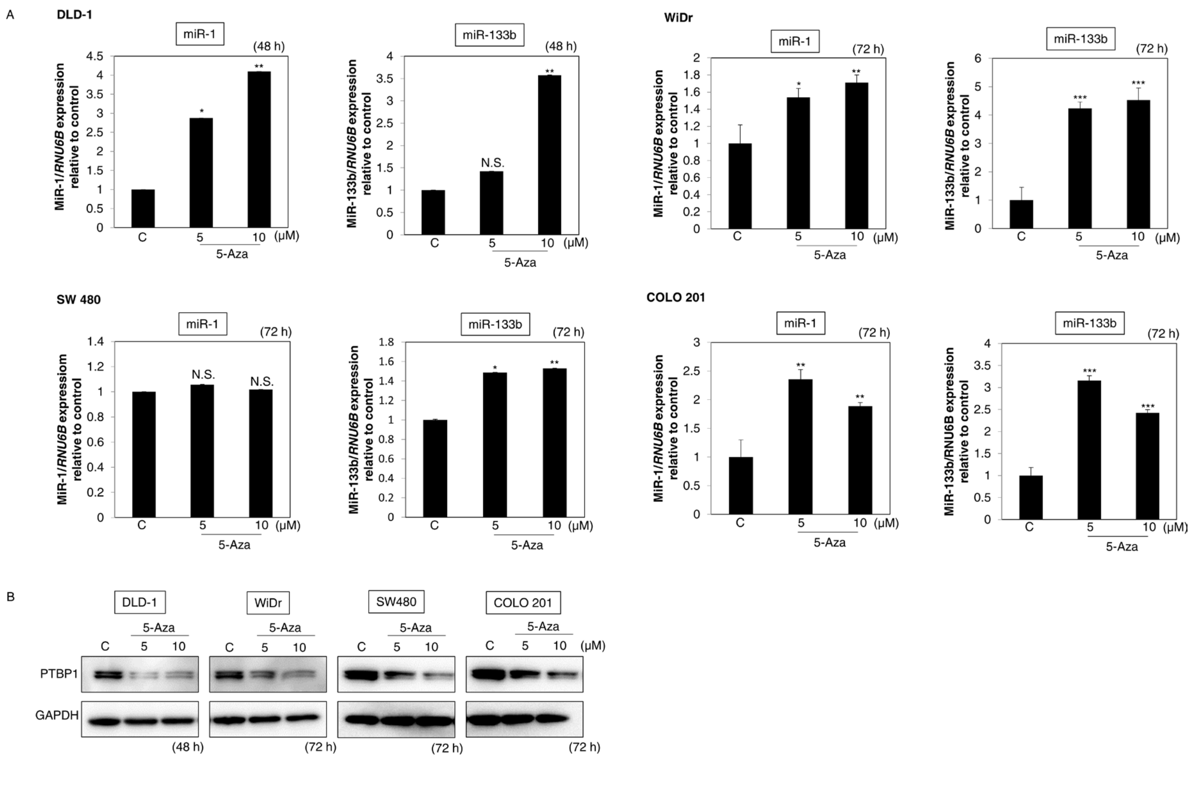

Supplementary Figure S2: (**A**) Expression levels of miR-1 and -133b in various colon cancer cells after treatment of them with 5-Aza (5 or 10 µM). The expression levels of the miRs were assessed at 48 h after the treatment in DLD-1 cells. Those in WiDr, SW480, and COLO 201 were assessed at 72 h. U6 was used as the control. (**B**) Expression of PTBP1 in 5-Aza-treated colon cancer cells. Assessment time was the same as in "A". GAPDH was used as the control. Results are presented as the mean ± SD; $*P < 0.05$; $**P < 0.01$; $***P < 0.001$.

A

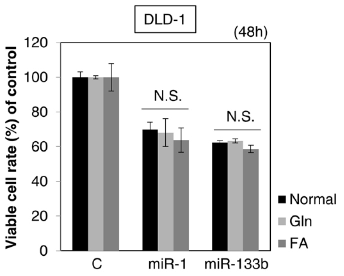

B

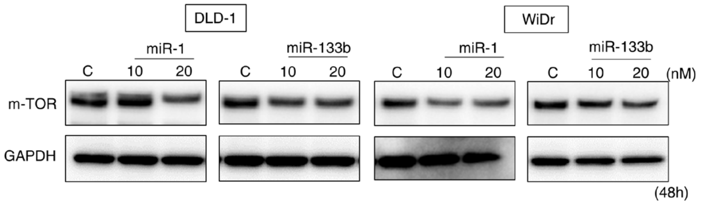

C

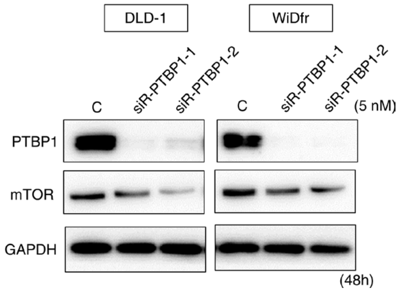

Supplementary Figure S3: (**A**) Effects of glutamine or fatty acid on cell growth of DLD-1 cells transfected with either miR. Glutamine or fatty acid was added at 5 h after the transfection with miR-1 and miR-133b (20 nM). We assessed the effects at 48 h after the addition of glutamine or fatty acid. Normal: RPMI-1640 with FBS, Gln: addition of glutamine to RPMI-1640 with FBS, FA: addition of fatty acid to RPMI-1640 with FBS (**B**) Protein expression of mTOR at 48 h after transfection with miR-1 or -133b (10, 20 nM). GAPDH was used as the control. (**C**) Protein expression of PTBP1 and m-TOR at 48 h after transfection of DLD-1 and WiDr cells with siR-PTBP1 (5 nM). GAPDH was used as the control. Results are presented as the mean ± SD; N.S., not statistically significant.

Supplementary Table S1: Clinicopathological features of colorectal tumors examined for PTBP1 expression (Figure 6E)

Case	Age	Sex[a]	Site[b]	Size[c]	Depth[d]	Stage[e]	PTBP1[g]	miR-1(T/N)[h]	miR-133b (T/N)[h]
1	49	F	R	70	SS	B	+	0.180	0.041
2	53	F	R	45	SS	B	+	0.030	0.018
3	68	M	R	65	SE	D	+	0.006	0.009
4	59	F	S	60	SE	C	+	0.570	0.293
5	73	M	R	40	MP	A	+	0.102	0.181
6	56	F	S	27	SS	C	+	0.138	0.143
7	67	M	S	55	MP	A	+	0.550	0.310
8	78	F	C	80	SI	C	+	0.050	0.017
9	52	M	S	25	MP	A	+	0.160	0.055
10	38	F	RS	95	MP	A	±	0.053	0.072
11	62	M	S	55	SS	D	+	0.130	0.150
12	60	M	R	68	SS	C	+	0.074	0.090
13	81	M	RS	50	MP	C	+	0.100	0.020
14	73	F	T	55	SS	B	+	0.254	0.190
15	49	M	R	50	SS	B	+	0.137	0.046
16	68	F	R	35	SS	B	+	0.487	0.100
17	92	M	S	25	MP	A	+	0.036	0.212
18	62	M	R	39	MP	A	+	0.299	0.134
19	83	F	D	28	SS	B	+	0.445	0.203
20	49	F	R	45	MP	A	+	0.017	0.015
21	64	F	A	29	SS	C	+	0.162	0.075
22	61	F	S	39	M	A	+	0.003	0.009
23	79	M	S	42	MP	C	+	0.028	0.021
24	18	F	A	65	SE	C	+	0.046	0.024
25	73	M	S	10	SM	A	+	0.270	0.120
**Case**	**Age**	**Sex[a]**	**Site[b]**	**Size[c]**	**Grade[e]**	**Stage**	**PTBP1[g]**	**miR-1(T/N)[h]**	**miR-133b (T/N)[h]**
26	50	F	S	20	H		+	0.160	0.070
27	54	F	S	20	L		+	0.210	0.250
28	66	F	A	15	L		+	0.270	0.340
29	79	M	S	15	L		+	0.060	0.060
30	73	M	R	10	H		+	0.330	0.140

We examined PTBP1 expression in 30 cases that were available for obtaining protein samples.
a M, male; F, female.
b Location of tumor; C, cecum; A, ascending colon; T, transverse colon; S, sigmoid colon; RS, rectosigmoid colon; R, rectum.
c Diameter in mm.
d M,mucosa; SM, submucosa; MP, Mucosa propria; SS, Subserosa; SE, Serosa exposure; SI, Serosa invasion.
e Dukes' system.
f L, low grade adenoma (mild and moderate atypia); H, high grade adenoma (sever atypia).
g +, overexpression; ±, no overexpression.
h The relative ratio of miR-1 or -133b (tumor tissue/normal adjacent tissue).
The ratio < 0.67 as down-regulation.
